# Maternal vaccination: A narrative review

**DOI:** 10.4102/sajid.v37i1.451

**Published:** 2022-09-26

**Authors:** Sahra Ashkir, Olive P. Khaliq, Mehreen Hunter, Jagidesa Moodley

**Affiliations:** 1 Women’s Health and HIV, Department of Obstetrics and Gynaecology, School of Clinical Medicine, University of KwaZulu-Natal, Durban, South Africa; 2 Department of Paediatrics and Child Health, Faculty of Health Sciences, School of Clinical Medicine, The University of the Free State, Bloemfontein, South Africa; 3 Division of Public Health Medicine, School of Public Health and Family Medicine, University of Cape Town, Cape Town, South Africa

**Keywords:** vaccinations, immunisation, pregnancy, foetus, neonates

## Abstract

**Background:**

Vaccinations in general are considered to be one of the greatest achievements in medicine, saving millions of lives globally.

**Aim:**

This narrative review highlights issues related to vaccination in pregnancy and provides information on those vaccines registered for use in pregnancy.

**Method:**

Published articles on vaccinations in pregnancy are included in this review. The search engines used included PubMed, Medline, Google Scholar, and ScienceDirect.

**Results:**

Vaccinations during pregnancy are more likely to be administered in high income countries (HICs) compared to low-income countries (LICs) due to easier access to healthcare services and better communicable disease awareness. Maternal and perinatal morbidity and mortality rates associated with infectious diseases are higher in LICs with access to maternal care services, infrastructure and hospital equipment lacking in these settings.

**Conclusion:**

Suitable vaccinations are recommended for use in pregnancy to prevent harm to women, their foetuses and newborns from some communicable diseases, and they have resulted in declines in maternal and infant morbidity and mortality. Furthermore, this review has shown that vaccination during pregnancy is not only safe for both the woman and her foetus but also effective. Therefore, health professionals and national governments should strongly consider approved vaccinations prior to or during pregnancy.

**Contribution:**

This review provides insight on the necessity of vaccination during pregnancy. In addition, it urges health professionals to inform patients of the importance of regular antenatal visits, and to receive the required vaccinations for a better health outcome.

## Introduction

Population-based vaccination programmes are cost-effective health interventions that reduce transmission of preventable diseases and save millions of lives.^1^ The practice of vaccinating children for the prevention of communicable diseases is a good example of this.^1,2,3^

Pregnancy and infancy increase susceptibility to infectious diseases, due to the immunological and physiological changes of pregnancy and childbirth.^1,2^ Vaccination uptake by pregnant women has varied greatly worldwide, with rates of < 16% in low-income countries (LICs) to levels of up to 90% for some vaccines in high-income countries (HICs).^2,4^ Maternal vaccination rates in developing countries declined greatly during the coronavirus disease 2019 (COVID-19) pandemic, possibly because of the lack of vaccine supplies, restricted population movements due to the lockdown and health facilities concentrating on providing emergency services.^4^

A few vaccines with adequate proof of safety are recommended during pregnancy.^3,5,6^ This review provides a synthesis of reviews of evidence supporting the use of vaccination in pregnancy.

## Methods

Relevant articles were identified using search engines such as Google Scholar, PubMed, Medline and ScienceDirect. The specific keywords that were used included ‘vaccinations’, ‘immunizations’, ‘pregnancy’ and ‘neonates’. The articles that were excluded were those that did not include pregnant women. The search had no restrictions in terms of years of publication, and original articles, narrative reviews, systematic reviews and meta-analyses were evaluated.

## The benefits of vaccination during pregnancy

Vaccination during pregnancy protects both the mother and her unborn baby from infections and the risk of intra-uterine infections. In addition, maternal vaccination provides protection for the baby in the first few months of life through placental transfer of neutralising antibodies (immunoglobulin G [IgG]) and/or immunoglobulin A (IgA) antibodies through breast milk, conferring passive immunity on the newborn during the first few months of life.^5,7,8,9,10^

Perinatal vaccination can be used as an alternative measure to prevent transmissible diseases. However, it is dependent on the infant’s immune system to generate antibodies, which may be difficult in the first few weeks of life.^2,5,7,8,9,10^ Furthermore, many vaccines are not administered to infants in the first six weeks of life due to the immature immune system, and if given, two doses of the vaccine may be required before full protection is achieved. A benefit of maternal vaccination is that it increases the transmission of maternal antibodies, thus overcoming infant vulnerability in the first six weeks of life.^5,7,9,10^

## Vaccine safety in pregnancy

Vaccines regarded to be safe during pregnancy include killed and inactivated virus vaccines, protein subunit vaccines, toxoid-containing vaccines and conjugated vaccines.^1,2,5^ There is no evidence that vaccinating pregnant or breastfeeding women with inactivated viral or bacterial vaccines or toxoids poses a risk to the foetus and/or the infant. Live vaccines, on the other hand, pose a risk of miscarriage and congenital infections and are therefore contraindicated in pregnancy.^2,3,6^ However, the American College of Obstetrics and Gynaecology is in favour of vaccinating women during pregnancy and has recommended that all pregnant women should be vaccinated at 27–36 weeks of gestation against tetanus, diphtheria ad pertussis.^11^ Unvaccinated pregnant women are reported to be at risk of maternal and foetal morbidity and mortality, with the risk decreasing in vaccinated women. Another important recommendation is that the healthcare professional (Professional Nurse and / Medical doctor) is required to monitor the patients regularly during their antenatal visits to ensure they receive all vaccines. In addition, women who fall pregnant or are pregnant during the influenza season are advised to get vaccinated against influenza, as an infection may cause adverse maternal and foetal outcomes.^11^ Presently, vaccines undergo phase 1 and 2 studies in nonpregnant women of reproductive age before clinical trials are performed in pregnancy. In circumstances in which there is an urgent need for a vaccine (such as the Ebola and COVID-19 pandemics), there can be undue delays in providing safety data to support the use of vaccination in pregnancy and the breast-feeding period.^7,8,9^ Vaccination trials on COVID-19 and Ebola excluded pregnant women, but they were included later. However, information on the safety and efficacy of these vaccinations was derived from women who were pregnant unknowingly and received the vaccination, as well as from those who volunteered to participate fully aware of their pregnancy status.^7,8,9^ This highlights the need for a coordinated approach for the inclusion of pregnant women in vaccine clinical trials, the need for developmental toxicology studies at the early point of the study and the use of vaccine platforms that are already known to be safe in pregnancy.^7,8,9,10^

## Increasing vaccine confidence among pregnant women, their families and partners

The COVID-19 pandemic has highlighted the low rates of vaccination in pregnancy globally.^5^ A review of studies investigated the factors that influenced vaccine uptake in pregnant women and found that the main determinants were awareness of the vaccine, disease severity and susceptibility, vaccine benefits, side effects and risk of harm during pregnancy and history of previous vaccination.^7,10,12,13^ Furthermore, Kilich et al. highlighted the fact that a pregnant woman’s decision on whether to accept vaccination is strongly determined by the recommendation of her healthcare professional.^12^ For this reason, healthcare professionals should not only offer but proactively recommend vaccination during pregnancy and provide ample time and opportunity for the woman to communicate her concerns to make an informed decision.^12^ While this can prove to be a burden to healthcare professionals who often work under stressful conditions, to improve the low uptake of vaccinations and to enhance the provision of holistic healthcare, healthcare providers should receive training on how to effectively counsel and provide care for women through informing them about the importance of vaccinations during pregnancy. Hoare et al. recommended that healthcare professionals should include a ‘motivational interview (MI) approach’ in counselling patients which includes a nonjudgemental collaborative interview with compassion and respect to overcome vaccine hesitancy.^13^ Another enabling measure to improve vaccine uptake is to create community-based platforms where individuals can be informed not only about the benefits of vaccination during pregnancy but about the safety profile as well.^13,14,15^ It is paramount in the world today that all forms of misinformation are countered with transparent, easily understood and evidence-based information that is widely disseminated to the public, in an effort to offset misleading, and often unfounded, fears surrounding healthcare interventions.

## Routinely recommended vaccines in pregnancy

### Influenza vaccination

Various studies have shown that pregnant women are at an increased risk of severe disease and death from seasonal influenza than nonpregnant women.^14,15,16,17,18^ This outcome was echoed more than a decade ago during the H1N1 influenza pandemic; approximately 7.2% of pregnant women were likely to be admitted to hospital compared to nonpregnant women and were found to have a disproportionally high risk of mortality.^7^ Recently, Dawood et al. reported that seasonal influenza during pregnancy resulted in miscarriages and low birth weight babies.^15^ In view of the increased risk of pregnant women to seasonal influenza, the World Health Organization (WHO) advised the use of the seasonal vaccination throughout the year.^1^ There is currently a lack of consensus on the gestational age at which the vaccine should be administered, because the aim of the vaccine is primarily for maternal not foetal benefit and should therefore occur as soon as possible during the flu season. Dawood et al. found that the incidence and severity of influenza was highest in the first trimester of pregnancy, which supports the recommendation to vaccinate in early pregnancy.^15^ However, receiving the vaccination during the third trimester is more beneficial to the foetus, because more antibodies are vertically transmitted through the placenta, thus increasing protection for the baby after birth for up to six months.^16,17,18^ In addition, breast-feeding facilitates the passage of antibodies to the infant.^9,16,17,18^ There is, however, a lack of robust data and the potential use of a second dose of the influenza vaccine during pregnancy needs investigation. The current recommendation by the WHO is that all pregnant women should be vaccinated for seasonal influenza and that the vaccine can be administered during any trimester of pregnancy.^1,14^ Despite the prioritisation of seasonal influenza vaccinations, coverage is < 16% in low- and middle-income countries such as South Africa (SA). However, recent studies have shown that dedicated antenatal influenza vaccination campaigns in selected sites in SA can reduce the severity of influenza while remaining cost-effective.^19,20^

### Pertussis vaccine

Pertussis or whooping cough is a highly contagious respiratory illness caused by the bacteria *Bordetella pertussis*. Pertussis resulted in high mortality rates before the discovery of a vaccine in the 1940s.^21,22^ Vaccination in childhood, adolescence and adulthood, excluding pregnant women, led to a substantial decrease in childhood deaths, but death of infants < 3 months old persisted.^23^

The pertussis vaccine is administered in combination with diphtheria and tetanus vaccines. The current combined vaccine against diphtheria, tetanus and pertussis (DTaP) includes an acellular pertussis vaccine consisting of highly purified individual B pertussis components. Tetanus, diphtheria and pertussis (Tdap) and tetanus and diphtheria (Td) are given to pregnant women, nonpregnant women, adults, adolescents and children over the age of seven, while DTap is given to children under the age of seven.^21,22,23^ Maternal immunisation is regarded as the safe option for protecting infants against pertussis from birth until the first vaccinations are administered at two months of age.^22^ Pertussis vaccination reports show no negative effects on the foetus regardless of maternal vaccination schedule and benefit the neonates two-fold through passive immunity and protection through contact immunisation.^21,24^ However, a major concern about maternal pertussis vaccination is blunting of the infant’s response to routine vaccinations. There is also a theoretical concern that providing this early immunity may interfere with the infant’s immune response to DTaP, resulting in the infant’s immune response to DTaP weakening.^23^ However, while researchers are still working to gain a better understanding of this problem, a recent study demonstrated that this interference did not appear to be a problem in terms of protecting infants.^23^

Therefore, vaccination during pregnancy by protecting a newborn outweighs the risk of blunting the infant’s response to diphtheria, tetanus, and acellular pertussis (DTaP). Because infants are most at risk of severe disease and death from pertussis before the age of 3 months, any protection that can be provided is critical.^23^ Tetanus, diphtheria and pertussis (Dap) vaccination can be postponed until later in pregnancy, avoiding concerns about foetal development interference or mistaken association with pregnancy loss, which is most common in the first trimester. A study found that pregnant women immunised with Dap between 27 and 30 + 6 weeks’ gestation conveyed the highest umbilical cord IgG to pertussis toxin (PT) and filamentous hemagglutinin (FHA).^23,24^ The Centers for Disease Control and Prevention (CDC) advises that Tdap be administered between 27 and 36 weeks of pregnancy in all cases; even if a pregnant woman has had a Tdap vaccine within the last 10 years, the vaccine should be administered again.^25^ Vaccination in late pregnancy has the added benefit of increasing maternal antibodies to the foetus when exchange of nutrients via the placenta is most efficient (from approximately 34 weeks gestation).^26^ Theoretically, this timing optimises levels of maternally derived antibodies in the newborn, resulting in longer protection.^27,28^

### Tetanus toxoid

In the early 1980s, neonatal tetanus was estimated to be responsible for over 500 000 neonatal deaths worldwide.^27^ Even though eradication is impossible due to environmental spores, the goal is disease elimination, which is defined as less than one case per 1000 live births.^3^ The CDC reported a significant decrease in neonatal deaths in 2019, which was approximately 34 700 worldwide.^29^ Complications arising from obstetric and postnatal umbilical-cord care practices, and maternal and neonatal tetanus remain public health issues in 48 countries, primarily in Asia and Africa.^30,31^ Without proper medical care and management of tetanus, mortality rates rise up to 100%, while survival of tetanus patients has improved significantly in hospitals (10% – 60%) with modern intensive-care facilities; however, such facilities are frequently unavailable in areas with the highest tetanus burden.^32,33,34^ Other factors reported to hinder tetanus vaccination in pregnant women include maternal age at their first pregnancy, educational status, socio-economic status, no access to health services and geographical status.^33,34^

Tetanus is caused by *Clostridium tetani* spores coming into contact with skin lesions after an injury.^33^ This may occur after delivery, abortions or miscarriages in women, while in neonates it may be due to the umbilical cord stump or unhygienic practices of an infected mother.^33^ Pregnant women are recommended to vaccinate and maintain continuous protection against tetanus, because this disease thrives in the soil and in faecal matter.^33^

The WHO advised that pregnant women should receive four doses of the combined tetanus toxoid and diphtheria toxoid (Tda) vaccinations against tetanus. The first dose should be given during the first antenatal visit, followed by the second one after four weeks for women who have no history of receiving the vaccination before. The third dose should be given six months after the second dose. Healthcare professionals are required to ensure that three doses are given two weeks prior delivery for the protection of the foetus during delivery. The last dose should be given a year after delivery.^35^

### COVID-19 vaccination

The severe acute respiratory syndrome coronavirus 2 (SARS-CoV-2) was discovered late November 2019 or December 2019 in Wuhan, China, and sparked the global pandemic of coronavirus disease 2019 (COVID-19).^36^ Physiologic adaptations and changes in immune regulation may increase the risk of morbidity and mortality in pregnant women with respiratory infections.^5,7,26^

Pregnant women are at an increased risk for severe illness from COVID-19 compared to age-matched women who are not pregnant.^5,19,26^ Severe illness includes an increased risk of respiratory failure, requiring hospitalisation, intensive care unit admissions and mechanical ventilation, or illness that results in death.^5,19,26^ Pregnant women are also at increased risk for stillbirths and preterm labour and in some cases, thrombotic events and pre-eclampsia.^37^ These high rates of maternal and perinatal complications associated with COVID-19 infections highlight the need for strategies to minimise risks. One such strategy is COVID-19 maternal vaccination.^5^ In cases where the COVID-19 vaccine is administered late, with the risk of preterm delivery, neonates can still receive protection through breastfeeding.^38^ Pregnant women who were vaccinated have active SARS-CoV-2 antibodies in their breast milk up to six weeks after vaccination, and the antibodies have been reported to show neutralising properties and may be efficient in terms of protecting neonates.^38^

Accumulating data provides evidence of both the safety and effectiveness of COVID-19 vaccination in women who had mRNA vaccines (Pfizer and Moderna) inadvertently.^39^ Based on this, professional maternal health organisations strongly recommend registered COVID-19 vaccines for pregnant women, because the benefits of vaccination for pregnant women, their foetuses and infants outweigh any known or potential risks. Furthermore, pregnant women with underlying conditions and those who provide health services such as physicians and nurses were prioritised to receive the COVID-19 vaccine, as these were a vulnerable group who were exposed to COVID-19-positive patients.^5,26,40^ However, maternal vaccination rates are relatively low, approximately 30% in both the United States and the United Kingdom.^39,40^ One of the reasons for this may be that studies investigating therapies for COVID-19 infections had initially excluded pregnant women and underscored the importance of including pregnant women and in clinical trials of treatment and vaccines.^39,40^ To improve vaccination rates in pregnant women, the CDC recommends ensuring culturally responsive and linguistically appropriate communication of vaccination benefits.

## Vaccines for pregnant women at risk

### Hepatitis B

Mother-to-child transmission (MTCT) occurs in about 60% of pregnancies linked with acute hepatitis B virus (HBV) infection at or near term.^36^ Preterm birth may be increased if acute hepatitis B is acquired in the final trimester. Sub-Saharan Africa (SSA), after Asia, carries the highest burden of HBV infection; therefore, vertical transmission of hepatitis B is still a major public health concern in this area.^36^ Antenatal screening for HBV infection is currently not performed in the public sector in SA, but it is the key step in identifying those women most at risk of transmitting infection and allowing the implementation of preventive strategies.^37^ High-risk populations, such as intravenous drug users and healthcare professionals, should be vaccinated. In pregnancy, the hepatitis B vaccine is a recombinant vaccine that is both safe and effective. An expedited vaccination schedule of three shots over one month is recommended for women who are particularly at risk.^41^

### Meningococcal

Meningococcal illness causes meningitis and septicaemia globally, with the largest incidence of disease occurring in children under the age of two, particularly young infants.^42^ Only two vaccinations, the bivalent A and C meningococcal polysaccharide vaccine (Menace-PS) and the quadrivalent polysaccharide vaccine (which protects against capsular groups A, C, W, and Y), have been used in pregnancy (Menace-PS).^42^ A study in Bangladesh showed that anti-Menace-PS geometric mean titres in cord blood were twice as high in babies delivered to vaccinated mothers compared to unvaccinated women after third-trimester immunisation with quadrivalent Menace A,C,W,Y,-PS.^43^ Although high-risk populations, such as military recruits, individuals with terminal complement component deficits and those with anatomic or functional asplenia, should be vaccinated on a routine basis, the presence of immunity would need to be confirmed first; this could be done by investigating previous history of the disease to evaluate the presence of antibodies prior to the vaccination.^14^ Travellers visiting locations where Neisseria meningitidis is prevalent or endemic, such as SSA, may benefit from the vaccination.^14^

## Vaccines under development for pregnant women

### Respiratory syncytial virus

The respiratory syncytial virus (RSV) is a common respiratory virus that causes a significant disease burden, with millions of hospitalisations and thousands of deaths in children under the age of five each year.^10^ Notably, approximately 45% of all severe RSV hospitalisations and deaths occur in infants under six months old. Considering the high number of infant deaths, manufacturing an RSV vaccination for neonates or for pregnant women would be beneficial. Currently, a particle-based vaccination against protein F for pregnant women is underway.^4,12^

### Group B streptococcus

Group B streptococcus (GBS) has been and remains a leading cause of maternal chorioamnionitis, puerperal endometritis and neonatal sepsis.^44^ Vaccination would significantly reduce the burden of infant GBS disease in SA and, according to the WHO, would be cost-effective.^45^ The standard practice for the management of GBS in pregnant women is intrapartum antibiotic prophylaxis.^10^ The process of developing the vaccination against GBS in pregnant women is currently ongoing.^10^

## Vaccinations in pregnancy: South African guidelines

In SA, all pregnant women aged 18 and above are included in immunisation at their antenatal clinics. Vaccination guidelines in 2016 state that the pregnant women should receive the tetanus vaccine despite neonatal tetanus being eliminated in the country. This is to sustain the elimination of the disease in neonates. It is recommended that women receive either Tetanus Toxoid (TT) or Tetanus and Diphtheria (Td).^46^

Recently, recommendations for women to receive the COVID-19 vaccine have been added to the existing guidelines. Pregnant women and lactating women are at risk of contracting COVID-19 more than nonpregnant women. Coronavirus disease 2019 infection during pregnancy compromises the health of both the mother and the unborn baby; therefore, the Vaccine Ministerial Advisory Committee (VMAC) recommended that all pregnant and lactating women aged 18 and above receive the COVID-19 vaccination. The Pfizer and the Johnson and Johnson vaccines are reported equally safe during pregnancy.^47^

## Summary of major findings and shortcomings

This narrative found that maternal vaccination is generally safe for the mother, her foetus and newborn. They are recommended for use in pregnancy globally, despite the fact that pregnant women have traditionally not been included in clinical and or animal studies. The COVID-19 pandemic has highlighted this shortcoming, and it is hoped that researchers will include consenting pregnant women in future trials, as some of these infectious diseases are associated with stillbirths and preterm labour; vaccination mitigates these adverse events.

Maternal vaccination should generally be offered after the first trimester, not only because of the fact that most studies have not included pregnant women but also because vaccination in the last two trimesters of pregnancy may lead to higher levels of antibodies being transferred to the foetus and during breast-feeding.

Other shortcomings of promoting maternal vaccination in developing countries are high financial costs of vaccinations, the lack of local manufacturing of the drugs and the need to spread information on the value about vaccinating pregnant women and neonates.

## Conclusion

Maternal vaccination is an effective public health measure for the protection against transmissible diseases and for the desired goal to reduce maternal and neonatal death. Despite this, maternal vaccination is an underutilised resource, especially in low- and middle-income countries. Therefore, it is imperative that maternal healthcare providers counsel pregnant women of the potential benefits of vaccination, not just for themselves but their foetuses and infants as well. They should proactively offer routinely recommended vaccinations. In addition, healthcare providers must be given proper education on how to guide pregnant women regarding the offer and recommendation of maternal vaccination.

Currently recommended vaccines globally include influenza, tetanus and pertussis–containing vaccines. Pregnant women and those planning on falling pregnant in the near future should consider taking the COVID-19 vaccination, given the accumulating data on its safety and effectiveness. Antenatal clinics and hospitals that manage pregnant women should provide booklets about communicable and infectious diseases in pregnancy and neonates and encourage all pregnant women and mothers to vaccinate against these diseases. Pregnant women should be included in clinical trials during the development of new vaccinations in the future.
